# Resistance to mTORC1 Inhibitors in Cancer Therapy: From Kinase Mutations to Intratumoral Heterogeneity of Kinase Activity

**DOI:** 10.1155/2017/1726078

**Published:** 2017-02-09

**Authors:** Seraina Faes, Nicolas Demartines, Olivier Dormond

**Affiliations:** Department of Visceral Surgery, Lausanne University Hospital, Pavillon 4, Av. de Beaumont, 1011 Lausanne, Switzerland

## Abstract

Targeting mTORC1 has been thoroughly explored in cancer therapy. Following encouraging preclinical studies, mTORC1 inhibitors however failed to provide substantial benefits in cancer patients. Several resistance mechanisms have been identified including mutations of mTOR and activation of alternate proliferation pathways. Moreover, emerging evidence discloses intratumoral heterogeneity of mTORC1 activity that further contributes to a reduced anticancer efficacy of mTORC1 inhibitors. Genetic heterogeneity as well as heterogeneous conditions of the tumor environment such as hypoxia profoundly modifies mTORC1 activity in tumors and hence influences the response of tumors to mTORC1 inhibitors. Intriguingly, the heterogeneity of mTORC1 activity also occurs towards its substrates at the single cell level, as mutually exclusive pattern of activation of mTORC1 downstream effectors has been reported in tumors. After briefly describing mTORC1 biology and the use of mTORC1 inhibitors in patients, this review will give an overview on concepts of resistance to mTORC1 inhibition in cancer with a particular focus on intratumoral heterogeneity of mTORC1 activity.

## 1. Introduction

Cancer is usually driven by an accumulation of genetic and epigenetic alterations which promote cell growth and immune escape. Hence, blocking these alterations represents a major treatment approach in cancer [1]. The initial success of imatinib in chronic myeloid leukemia demonstrated the feasibility of such an approach which was further extensively developed in cancer therapy [2]. Patient stratification, based on cancer genotyping, aimed at identifying the driving forces in a tumor, should help chose the appropriate treatment. However, over years, several drawbacks were identified that limit the efficacy of this approach. In particular, tumor heterogeneity emphasizes the complexity of cancer cells, where frequently more than one driving force for tumor progression coexists heterogeneously in cancer [3]. Furthermore, not only a random accumulation of mutations induces tumor heterogeneity, but also in fact variable tumor environmental traits add another level of complexity to this process [4]. In addition to tumor heterogeneity, development of cellular resistance to a specific treatment represents a major hurdle to targeted therapies in cancer. Several resistance mechanisms have been identified, including secondary alterations in the target and activation of bypass mechanisms [5]. Hence, despite promising preclinical studies, most targeted therapies have failed to provide prolonged benefits in cancer patients.

In the context of personalized therapies in cancer, the mammalian target of rapamycin complex-1 (mTORC1) represents a fascinating topic that has been extensively explored. mTORC1 perfectly reflects the problem of targeted therapies, being conceptually and preclinically a promising target but displaying only limited efficacy if targeted by mTORC1 inhibitors in clinical trials. Several causal factors for a limited efficiency of mTORC1 inhibitors have been identified and will be described in this review with a particular focus on the intratumoral heterogeneity of mTORC1 activity.

## 2. mTORC1 and Cancer

**Figure 1:**
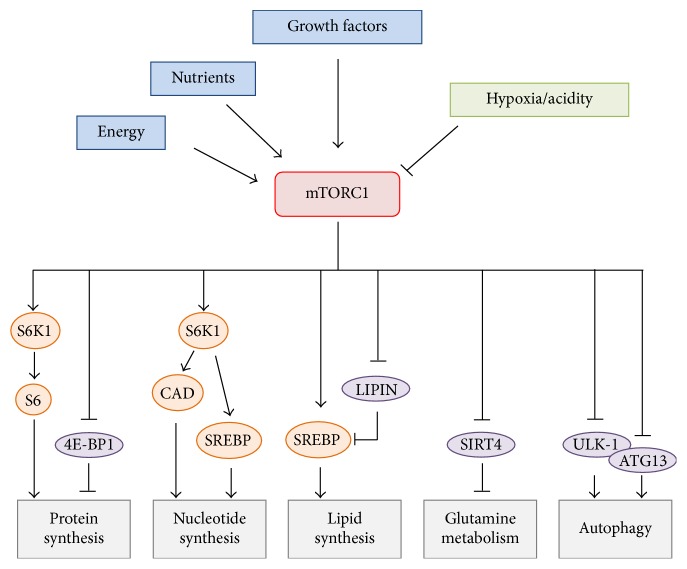
mTORC1 regulates cellular anabolic processes. mTORC1 is activated by growth promoting conditions including energy, nutrients, and growth factors. In contrast, unfavorable conditions such as hypoxia or acidity inhibit mTORC1. Once activated, mTORC1 promotes key anabolic processes that lead to cell growth. In addition, mTORC1 inhibits autophagy. A nonexhaustive list of downstream effectors of mTORC1 is displayed.

mTORC1 is an ubiquitously expressed protein complex that controls cell growth by inducing protein and nucleotide synthesis, ribosome biogenesis, and lipogenesis and by blocking autophagy (Figure 1) [6, 7]. mTORC1 is able to sense environmental signals including growth factors and nutrients and initiates cell growth in favorable environmental conditions. In contrast, unfavorable conditions such as acidity and hypoxia, which are frequently encountered in the tumor microenvironment, inhibit mTORC1 activity [8, 9]. Among the different signaling pathways that transmit extracellular signals to mTORC1, oncogenic PI3K/AKT and RAS/RAF/MEK/MAPK pathways have been well characterized. Activation of these pathways leads to the phosphorylation and inhibition of TSC2 which, in association with TSC1, forms a protein complex that inhibits mTORC1 [10–12]. Of note, mutations in the* TSC1* or* TSC2* gene are responsible for the tuberous sclerosis complex (TSC), a disease characterized by a variety of benign tumors found in multiple organs including the brain, kidneys, liver, heart, and lungs [13]. Following activation, mTORC1 phosphorylates a variety of substrates such as S6K1 and 4E-BP1, leading overall to an anabolic cellular response and resulting in cell growth and proliferation [6, 14, 15].

Since mTORC1 controls cell growth, it represents a potential target in cancer therapy. mTORC1 hyperactivation is furthermore frequently observed in sporadic cancers, either through activating mutations of upstream effectors of mTORC1 or through activating mutations of mTOR itself [16–18]. Additionally, enhanced activation of mTORC1 is observed in hamartoma syndromes including Peutz-Jeghers syndrome, Cowden disease, and TSC that are characterized by the development of benign tumors and mutations in tumor-suppressor genes that negatively regulate mTORC1 activity [19].

Besides mTORC1, another protein complex called mTORC2 exists [20]. In contrast to mTORC1, less is known about the functions of mTORC2. It is mainly activated by growth factors and it preferentially phosphorylates and activates proteins belonging to the AGC protein kinases family including AKT (Ser 473) and SGK1 (Ser 422). As such, mTORC2 also promotes tumor growth, and blocking its activity displays antitumoral effects in various preclinical models [21–25]. Nevertheless, for the purpose of this review, we will primarily focus on the effects of mTORC1 inhibition in cancer.

Different options exist to target mTORC1. By now, three generations of mTORC1 inhibitors have been developed [26]. Rapamycin and its derivatives termed rapalogs are the first generation of mTORC1 inhibitors. They inhibit mTORC1 by binding together with FKBP12 to the FRB domain, a domain adjacent to the kinase domain of mTOR, limiting the access of substrates to the active kinase site [27, 28]. Of note, rapalogs only incompletely block mTORC1, as, for example, mTORC1 phosphorylates the Thr-37 and Thr-46 sites of 4E-BP1 that are rapamycin insensitive [29]. Besides rapalogs, a second generation of mTOR inhibitors, termed ATP-competitive inhibitors of mTOR, has been generated. They inhibit the kinase domain of mTOR and therefore block both mTORC1 and mTORC2 [30, 31]. Also, compared to rapalogs, they exhibit a more profound inhibition of mTORC1. Due to kinase similarities between mTOR and other kinases such as PI3K, some of these kinase inhibitors of mTOR also block PI3K in addition to mTORC1 and mTORC2 [30]. Finally, recently, mTOR resistance mutations to both rapalogs and kinase inhibitors of mTOR have been identified [32]. To overcome this resistance, a third generation of mTOR inhibitor was developed, called Rapalink, containing rapamycin crosslinked with a kinase inhibitor of mTOR in the same molecule [32].

## 3. Lessons Learned from the Use of mTORC1 Inhibitors in Clinic

Rapalogs have been routinely used in clinic, in particular as immunosuppressive agents. In contrast, ATP-competitive inhibitors of mTOR have not yet been approved and are tested in clinical trials. Rapalink is only in its experimental development. Hence, information regarding mTORC1 inhibition in patients has mostly been gathered from rapalogs. Overall, rapalogs exhibit antiproliferative effects in untransformed cells. Indeed, they effectively block T-cell proliferation in transplanted patients [33, 34]. Similarly, significant antitumor responses to rapalogs were observed in benign tumors of TSC [35, 36]. For instance, 75% of patients presenting a subependymal giant cell astrocytoma had more than 30% volume reduction of their lesions at 6 months of treatment [37]. Similar effects were noted in TSC patients with other types of benign tumors, including renal angiomyolipoma, fascial angiofibroma, lymphangiomyomatosis, cardiac rhabdomyoma, and retinal astrocytic hamartoma [35]. Tumors however regrew with cessation of therapy, demonstrating that rapalogs are rather cytostatic than cytotoxic.

The antitumor efficacy of rapalogs was more disappointing in sporadic cancers [21, 38]. Overall, rapalogs did not provide any long lasting benefits, increasing median overall survival by only a few months. Nevertheless, rapalogs are currently approved for the treatment of renal cell carcinoma [39, 40], advanced pancreatic neuroendocrine tumors [41], postmenopausal hormone receptor-positive advanced breast cancer in combination with exemestane [42], and advanced nonfunctional neuroendocrine tumors of the lung or gastrointestinal tract [43]. In Europe, they are further approved for the treatment of relapsed or refractory mantle cell lymphoma [44]. Most of the time rapalogs cause disease stabilization and fail to induce tumor regression, further highlighting that rapalogs are cytostatic. Hence, although several clinical trials are still investigating the anticancer efficacy of rapalogs, it is very unlikely that rapalogs will cure cancer.

Clinical trials with rapalogs revealed that blocking mTORC1 is associated with nonnegligible side effects [45, 46]. Since mTORC1 is ubiquitously expressed, blocking its activity in cancer therapy lacks specificity. The side effects include a variety of dermatological, metabolic, renal, hematological, and respiratory toxicities that often require dose reduction. These side effects are mostly moderate but can also be life-threatening in the case of pneumonitis. By generating discomfort, mTORC1 inhibitors are also responsible for an important prevalence of drug discontinuation [45]. Toxicities of mTORC1 inhibitors should hence be kept in mind, particularly when combined therapies are considered.

## 4. Limitations of mTORC1 Inhibitors in Cancer Therapy

**Figure 2:**
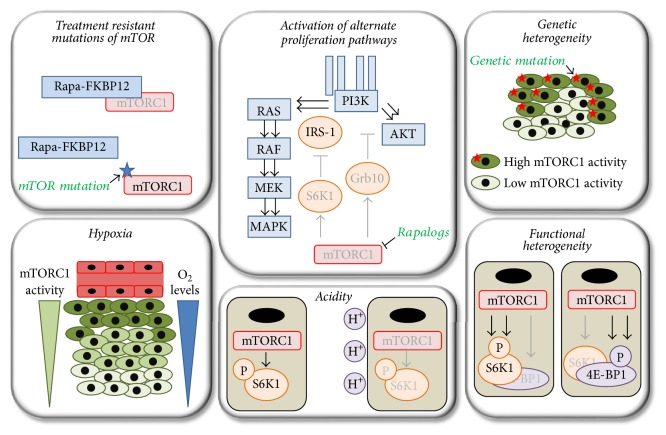
Factors impeding the anticancer efficacy of rapalogs.* Treatment resistant mutations of mTOR*: mutations of the FRB domain of mTOR block the binding of rapamyicn-FKBP12 to mTOR and impede the anticancer efficacy of rapalogs. Mutations conferring a hyperactive state of mTOR that resists ATP-competitive inhibitors of mTOR have also been reported.* Activation of alternate proliferation pathways*: upon inhibition of mTORC1, negative feedback loops are abolished, leading to an overactivation of PI3K/AKT and RAS/RAF/MEK/MAPK pathways that counteract the anticancer efficacy of rapalogs.* Genetic heterogeneity*: cancer cells harboring genetic mutations that lead to mTORC1 overactivation coexist with cancer cells displaying low mTORC1 activity. The latter exhibit an mTORC1-independent growth and are therefore resistant to mTORC1 inhibition.* Hypoxia*: hypoxia inhibits mTORC1; hence mTORC1 activity is reduced in hypoxic tumor regions, and these regions are resistant to mTORC1 inhibitors.* Acidity*: acidity inhibits mTORC1 activity in cancer cells in vitro, resulting in mTORC1-independent cancer cell growth.* Functional heterogeneity*: mTORC1 activity towards its downstream effectors is heterogeneous, where cancer cells displaying S6K1^high^/4E-BP1^low^ and S6K1^low^/4E-BP1^high^ phosphorylation patterns coexist in the same tumor. Rapalogs do not completely block mTORC1 activity with mTORC1-mediated 4E-BP1 phosphorylation being in part resistant to rapalogs. Beige squares and green ovals symbolize cancer cells and black ovals symbolize nuclei. Dark green: high mTORC1 activity; light green: low mTORC1 activity. Functionally active components of intracellular signaling pathways are displayed in black writing; functionally inactive components of intracellular signaling pathways are displayed in grey writing.

Despite a significant efficacy in preclinical models, the clinical tumor response to rapalogs is modest. Several factors might explain this limited impact in clinical applications [47]. As mentioned above, rapalogs provide only an incomplete inhibition of mTORC1 [48]. To overcome this limitation, ATP-competitive inhibitors of mTORC1 were generated that completely block mTORC1. These inhibitors further block mTORC2, which represents an additional advantage over rapalogs. In vitro and in vivo experiments demonstrated a stronger anticancer efficacy of these second generation inhibitors compared to rapalogs [49, 50]. ATP-competitive inhibitors of mTOR are currently being tested in clinical trials, but so far, an ample antitumor response has not been reported [51]. In addition, several other limitations of targeting mTORC1 in cancer therapy have been described, including treatment resistant mutations of mTOR, activation of alternate proliferative signaling pathways, and intratumoral heterogeneity of mTOR activity (Figure 2). These will be further discussed here.

## 5. Treatment Resistant Mutations of mTOR

Secondary genetic alterations of the targeted kinase represent a classic drug resistance mechanism and have been identified in a variety of tumors of patients treated with kinase inhibitors [5, 52–54]. Similarly, acquired resistance mutations of cancer cells exposed to mTORC1 inhibitors have been reported [32]. Treatment of MCF-7 breast cancer cell line with rapamycin or an ATP-competitive inhibitor of mTOR for three months led to the emergence of resistant colonies. Genomic sequencing revealed that rapamycin resistant clones harbored mutations in the FRB domain of mTOR. In contrast, the ATP-competitive inhibitor resistant clone contained an mTOR mutation located in the kinase domain. Both types of mutations were responsible for drug resistance [32]. FRB domain mutation disrupted the interaction between mTOR and FKBP12-rapamycin, consistent with data generated in yeast (Figure 2) [55, 56]. Mutations that conferred resistance to ATP-competitive inhibitors of mTOR did not alter binding of the drug to mTOR but generated a hyperactive state of the kinase. Interestingly, this type of mutation induces a hyperactive state of both mTORC1 and mTORC2, highlighting that resistance to ATP-competitive inhibitors of mTOR can occur from both mTORC1 and mTORC2. More importantly, both types of mutations have been detected in untreated patients, suggesting that certain types of cancer are intrinsically resistant to mTORC1 inhibitors [32]. A resistance mutation of the FRB domain has also been shown to be acquired under treatment with rapalogs in human. Indeed, resistance developed in a patient treated with the rapalog RAD001 for metastatic anaplastic thyroid carcinoma after an initial 18-month response [57]. Whole genome sequencing revealed that this tumor contained an FRB domain mutation after treatment that was not present initially. Of note, it also identified a nonsense mutation of TSC2 which could explain the initial high sensitivity of this tumor to RAD001 [57].

On the contrary, some of the identified hyperactivating mutations of mTOR are associated with increased sensitivity to rapamycin, suggesting that cancer cells harboring such mutations have an mTOR dependent proliferation pattern [18]. In these patients, such mutations could serve as biomarker in predicting cancer response to mTORC1 inhibitors. Consistent with this observation, a patient with metastatic urothelial carcinoma containing an mTOR hyperactivating mutation experienced a complete radiological response that lasted for 14 months after initiation of a treatment with RAD001 in combination with the tyrosine kinase inhibitor pazopanib [17].

## 6. Activation of Alternate Proliferative Signaling Pathways following mTORC1 Inhibition

mTORC1 belongs to a complex network of regulatory feedback loops responsible for limiting the proliferative signals transmitted by upstream effectors once mTORC1 gets activated (Figure 2) [47, 58, 59]. For instance, mTORC1/S6K1 mediated insulin receptor substrate-1 (IRS-1) phosphorylation enhances its degradation with subsequent disruption of PI3K/AKT signaling [60–62]. Similarly, mTORC1 activation leads to platelet derived growth factor receptors *α* and *β* degradation and attenuation of PI3K/AKT activity [60]. Likewise, mTORC1 stabilizes Grb10, leading to the inhibition of PI3K/AKT and MEK/MAPK pathways [63, 64]. mTORC1 activation also leads to a direct reduction of mTORC2 activity. For instance S6K1 phosphorylates Sin1, a component of mTORC2, at Thr 86 and Thr 398, resulting in the dissociation of Sin1 from mTORC2 and suppression of mTORC2-mediated activation of AKT [65, 66]. The observation that S6K1 phosphorylates rictor (Thr 1135), another component of mTORC2, suggests that additional feedback mechanisms exist between mTORC1/S6K1 and mTORC2/AKT; their functional relevance needs however to be further characterized [67, 68]. As a consequence, blocking mTORC1 results in the activation of proliferative pathways that will counteract the anticancer efficacy of mTORC1 inhibitors [69, 70]. For example, increased phosphorylation of AKT (Ser 473) was noticed in tumor metastasis of patients treated with rapalogs [71, 72]. Similar findings were reported in glioblastoma patients treated with rapamycin; an increased AKT phosphorylation (Ser 473) was further associated with a shorter time to progression [73]. Activation of upstream proliferative pathways by rapalogs is not limited to AKT, as rapalogs were also shown to increase MAPK activity. Indeed, tumor biopsies before and after RAD001 treatment demonstrated that MAPK phosphorylation (Thr 202/Tyr 204) was increased after treatment [74]. In experimental settings, blocking AKT or MAPK potentiated the anticancer efficacy of rapalogs, underlining that rapalogs-mediated AKT and MAPK overactivation dampens their efficacy [71, 74].

Second generation of mTORC1 inhibitors also abrogates negative feedback loops. As a consequence, their antitumor activity is also reduced by the activation of upstream pathways [69]. Like rapalogs, mTOR kinase inhibitors increase PI3K activity. Hence, AKT Thr308 phosphorylation is reinforced and sufficient to promote AKT signaling despite the loss of AKT Ser473 phosphorylation mediated by the inhibition of mTORC2 by kinase inhibitors of mTOR [75]. Surprisingly, restoration of AKT signaling following treatment with a dual PI3K/mTOR kinase inhibitor has also been reported [76]. In this case, PI3K-independent mechanisms are responsible for AKT phosphorylation and activity [77]. Besides AKT, an overactivation of MAPK by kinase inhibitors of mTOR has also been described [78, 79]. Interestingly, MAPK activation by kinase inhibitors of mTOR was independent of PI3K, arguing for a different loop between mTORC1 and MAPK apart from the previously identified loop with rapalogs [74]. Furthermore, since the feedback loop does not involve PI3K, dual PI3K/mTOR inhibitors likewise increase MAPK activity [69].

## 7. mTORC1 Activity Is Heterogeneous in Cancer

Recently, an old paradigm, namely, tumor heterogeneity, has been revisited in cancer biology [80, 81]. Emerging evidence demonstrates that genetic heterogeneity exists at the single cell level in cancer and therefore participates in resistance to a specific treatment [82, 83]. Like other signaling pathways, heterogeneous mTORC1 activity in a tumor has been reported. For example, immunohistochemical staining of human breast cancer for phospho-4E-BP1 (Thr 70) and phospho-S6 (Ser 240/244), as markers of mTORC1 activity, shows a marked heterogeneity among cancer cells, exhibiting either a strong or a weak staining in the same tumor [84, 85]. Moreover, genome sequencing of different regions of a human renal cell carcinoma revealed that a kinase domain mutation of mTOR was not present in every tumor region (Figure 2) [86]. Tumor cells displaying this mutation showed increased staining of phospho-S6 (Ser 235/236) and phospho-4EBP1 (Thr 37/46), suggesting that the mutation conferred increased activity to mTORC1. This study further showed that genetic intratumor heterogeneity is associated with a functional heterogeneity of mTORC1 kinase activity and possibly sensitivity to mTORC1 inhibitors [32]. Of note, this mTOR mutation was not detected in tumor metastases, further highlighting heterogeneity between the primary tumors and metastases [86]. This latter observation echoes well with a previous study that showed a poor concordance of mTORC1 activity in primary breast tumors and their corresponding metastases, as demonstrated by immunohistochemical staining of phospho-4E-BP1 (Ser 65) [87]. Other mutations of mTOR have further been identified, but their spatial distribution in a tumor has not yet been revealed [17, 18, 88, 89]. Genetic tumor heterogeneity has also been reported for proteins that belong to signaling pathways that lead to mTORC1 activation, such as PI3K/AKT and Ras/Raf/MEK/MAPK pathways. A high discrepancy in PI3K mutations between primary breast tumors and their metastases was reported [90]. More importantly, wild-type PI3K, mutated PI3K (H1047R), and mutated PI3K (E542K) were all detected in separate tumor regions of the same primary tumor. Similarly, intratumoral heterogeneity for K-Ras has been detected in samples of human colorectal cancer [91, 92]. Although mTORC1 activity was not specifically determined in these studies, one can speculate that the heterogeneous activation of upstream effectors of mTORC1 contributes to an intratumoral heterogeneity of mTORC1 activity.

Interestingly, emerging evidence depicts that mTORC1 activity towards its downstream effectors is also heterogeneous in tumors (Figure 2) [93, 94]. Using a multiplexed fluorescence microscopy method in human colorectal cancer, it was demonstrated that phosphorylation of S6 (Ser 235/236) and phosphorylation of 4E-BP1 (Thr 37/46) rarely occur in the same cancer cell but rather show mutual exclusivity [93]. Although crosstalk to other pathways cannot be fully excluded, this study supports a functional variation of mTORC1 to its downstream targets. Most probably, different mechanisms, which still need to be identified, regulate mTORC1 signaling to S6 and 4E-BP1. Additionally, since rapalogs do not block mTORC1-mediated 4E-BP1 phosphorylation (Thr 37/46), cancer cells displaying a phospho-S6^low^/phospho-4E-BP1^high^ pattern might be intrinsically resistant to rapalogs despite the presence of mTORC1 activity in these cells.

Tumor heterogeneity is not restricted to genomic evolution but also includes other types of heterogeneity [3, 94]. For example, physicochemical properties of the tumor microenvironment, such as oxygen levels and pH values, vary considerably between tumor regions and therefore contribute to heterogeneity and influence response to treatment. Indeed, regions of hypoxia are frequently present in tumors due to a high rate of cancer cell proliferation combined with a reduced perfusion caused by structural abnormalities of blood vessels [95]. Not surprisingly, as mTORC1 is inhibited by hypoxia in vitro, several studies have demonstrated that mTORC1 activity is reduced or absent in tumor hypoxic regions (Figure 2). For instance, mTORC1 activity as evidenced by immunostaining of the phosphorylated form of S6 ribosomal protein (Ser 235/236) negatively correlated with pimonidazole staining, a marker of hypoxia in HT29 tumor xenografts and MC-38 tumor allografts [96]. Similarly, in CAKI-1 tumor xenografts, tumor regions that stained positive for HIF-1*α* had no or little phospho-S6 staining (Ser 235/236) [97]. Furthermore, in human head and neck squamous cell carcinoma, the staining pattern of cancer cells for the hypoxia-regulated glucose transporter Glut-1 was inversely correlated with phospho-S6 staining (Ser 235/236) [98]. In addition, treatment of Rag2M mice bearing MCF-7 tumor xenografts significantly decreased tumor hypoxia and increased mTORC1 activity as demonstrated by Western blot by reduced HIF-1*α* expression and increased S6K1 phosphorylation (Thr 412), respectively [99]. In a mouse model of pancreatic neuroendocrine tumors, phospho-S6 staining (Ser 235/236) was restricted to the normoxic regions of the tumor following treatment with the antiangiogenic compounds sunitinib or axitinib [100]. Finally, in patient-derived renal cell carcinoma tumor xenografts, phospho-S6 staining (Ser 235/236) was predominantly observed around tumor blood vessels and colocalized with the lactate transporter MCT-1 that is specifically expressed in normoxic tumor regions [101]. Taken together these studies suggest that mTORC1 activity is predominantly found in the normoxic region of tumors and further underline that hypoxia, as an environmental signal, is able to directly influence signaling pathways such as mTORC1. They further highlight that regional variations accounting for intratumoral heterogeneity are not only a consequence of random acquisition of mutations. In other words, tumor heterogeneity is not limited to clonal differences [3].

Since hypoxic tumor cells still actively participate in tumor progression, these reports further suggest that tumor regions displaying low levels of oxygen grow independently of mTORC1 and are therefore insensitive to mTORC1 inhibition. Consistent with this hypothesis, it was found that, whereas rapamycin reduced cancer cell proliferation in nonhypoxic tumor area, it had no effect in hypoxic tumor regions, highlighting that rapamycin exerts a tumor region selective antiproliferative effect [96]. The observation that rapamycin decreased cancer cell proliferation in the outer well vascularized tumor regions but not in the hypovascular part of tumors further substantiates this hypothesis [102]. Hence, these observations suggest that, in cancer therapy, mTORC1 inhibitors should be combined with treatments targeting the hypoxic tumor compartment. In this context, blocking carbonic anhydrase IX, which is specifically upregulated by hypoxia in tumors and participates in tumor progression, represents a promising approach [103]. In fact, the inhibition of carbonic anhydrase IX by RNA interference or acetazolamide, a nonspecific inhibitor of carbonic anhydrases, increased the anticancer efficacy of rapamycin in cancer mouse models [96].

It is important to note that hypoxia not necessarily leads to mTORC1 inhibition. Certain cancer cells are able to maintain high levels of mTORC1 activity in hypoxic tumor regions, which adds another level of complexity to the relationship between mTORC1 and hypoxia [104]. The molecular mechanisms implicated in hypoxia-mediated mTORC1 inhibition have to some extent been characterized. They involve HIF1*α*-induced REDD1 expression [105, 106]. In turn, REDD1 inactivates mTORC1 activity in a TSC1/TSC2 dependent mechanism. The Ataxia Telangiectasia Mutated (ATM) protein also contributes to hypoxia-mediated mTORC1 inhibition by phosphorylating HIF1*α* which is necessary to induce REDD1 expression [104]. Hence, tumor cells harboring disrupted components of this signaling pathway, such as low levels of ATM, display a paradoxically elevated mTORC1 activity in hypoxic tumor regions [104]. In this context, hyperactivating mutations of mTOR also induce resistance to the inhibition mediated by high levels of REDD1 and might contribute to the maintenance of high levels of mTORC1 activity under hypoxia [89].

Besides hypoxia, tumors also frequently harbor regions of low pH [107]. Indeed, tumor cells preferentially perform glycolysis despite the presence of oxygen, hence inducing acidity and creating a hostile acidic tumor microenvironment [108]. Recent in vitro studies support a role of acidity in the inhibition of mTORC1 [9, 109, 110]. Exposing cancer cells to acidic pH leads to the downregulation of mTORC1 activity (Figure 2). Hence, like for hypoxia, cancer cells cultured in acidic conditions prosper independently of mTORC1. Future studies are however needed to characterize the molecular mechanisms involved in acidity-mediated mTORC1 inhibition and address whether acidity contributes to the mTORC1 activity heterogeneity in tumors.

## 8. Conclusions

Despite promising anticancer results in preclinical models, mTORC1 inhibition did not meet the expectations in clinical trials. Most trials were however performed in advanced cancer, possibly reducing the chance of success of mTORC1 inhibitors. Nevertheless, several limiting factors have been identified that help understand the weak clinical response. In fact, emerging evidence suggests a particularly heterogeneous activity of mTORC1 in tumors as an important limiting factor for the efficacy of mTORC1 inhibitors. Several elements contribute to this heterogeneity including genetic and functional heterogeneity as well as tumor hypoxia. Although tumor genetic screenings identified mTOR and TSC mutations that are associated with long term therapeutic benefits, most tumors eventually relapse within one or two years. Future therapy approaches will have to acknowledge and approach tumor heterogeneity, as mTORC1 inhibitors in monotherapy have failed to cure cancer.

## Abbreviations

4E-BP1: Eukaryotic translation initiation factor 4E-binding protein 1
AGC: Protein kinase A, G, and C families
ATG13: Autophagy-related protein 13
ATM: Ataxia telangiectasia mutated protein
ATP: Adenosine triphosphate
CAD: Carbamoyl-phosphate synthetase 2
FKBP12: FK506 binding protein 12
FRB: FKBP-rapamycin binding domain
GLUT1: Glucose transporter 1
GRB10: Growth factor receptor-bound protein 10
HIF1*α*: Hypoxia-inducible factor 1*α*
K-Ras: Kirsten rat sarcoma
MAPK: Mitogen-activated protein kinase
MC-38: Murine colon adenocarcinoma cell line 38
MCF-7: Michigan Cancer Foundation-7
MCT-1: Monocarboxylate transporter 1
MEK: MAP (mitogen-activated protein) kinase/ERK (extracellular signal-regulated kinase)
mTOR: Mechanistic target of rapamycin
mTORC1: Mechanistic target of rapamycin complex 1
mTORC2: Mechanistic target of rapamycin complex 2
PI3K: Phosphatidylinositol 3-kinase
REDD1: Protein regulated in development and DNA damage response 1
RNA: Ribonucleic acid
S6K1: P70 ribosomal protein S6 kinase 1
SGK1: Serum- and glucocorticoid-inducible protein kinase 1
Sin1: Stress activated protein kinase interacting protein 1
SREBP: Sterol regulatory element-binding proteins
TSC1: Tuberous sclerosis complex 1
TSC2: Tuberous sclerosis complex 2
ULK-1: UNC-51-like kinase 1.
